# The Impact of Hybrid Bionanomaterials Based on Gold Nanoparticles on Liver Injury in an Experimental Model of Thioacetamide-Induced Hepatopathy

**DOI:** 10.3390/biom15081068

**Published:** 2025-07-24

**Authors:** Mara Filip, Simona Valeria Clichici, Mara Muntean, Luminița David, Bianca Moldovan, Vlad Alexandru Toma, Cezar Login, Şoimița Mihaela Suciu

**Affiliations:** 1 Department of Physiology, “Iuliu Hatieganu” University of Medicine and Pharmacy, 1-3 Clinicilor Street, 400006 Cluj-Napoca, Romania; filip.mara@elearn.umfcluj.ro (M.F.); cezar.login@umfcluj.ro (C.L.); mihaela.suciu@umfcluj.ro (Ş.M.S.); 2 Department of Cell and Molecular Biology, “Iuliu Hatieganu” University of Medicine and Pharmacy, 6 Louis Pasteur Street, 400349 Cluj-Napoca, Romania; muntean.mara@elearn.umfcluj.ro; 3 Research Centre for Advanced Chemical Analysis, Instrumentation and Chemometrics, Faculty of Chemistry and Chemical Engineering, Babes-Bolyai University, 400347 Cluj-Napoca, Romania; luminita.david@ubbcluj.ro (L.D.); bianca.moldovan@ubbcluj.ro (B.M.); 4 Department of Molecular Biology and Biotechnology, Faculty of Biology and Geology, Babes-Bolyai University, 5-7 Clinicilor Street, 400006 Cluj-Napoca, Romania; vlad.toma@ubbcluj.ro; 5 Institute of Biological Research Cluj, National Institute of Research and Development for Biological Sciences, 48 Republicii Street, 400015 Cluj-Napoca, Romania

**Keywords:** gold nanoparticles, natural extract, hepatoprotective agent, thioacetamide, oxidative stress, inflammation

## Abstract

The present study aimed to evaluate the therapeutic benefits of a hybrid material based on gold nanoparticles and natural extracts on an experimental model of thioacetamide-induced (TAA) liver injury in rats. The nanomaterials were synthesized using a green method, with *Cornus sanguinea* L. extract as a reducing and capping agent (NPCS), and were then mixed with *Vaccinium myrtillus* L. (VL) extract in order to achieve a final mixture with enhanced properties (NPCS-VL). NPCSs were characterized using UV–vis spectrophotometry and transmission electron microscopy (TEM), which demonstrated the formation of spherical, stable gold nanoparticles with an average diameter of 20 nm. NPCS-VL’s hepatoprotective effects were evaluated through an analysis of oxidative stress, inflammation, hepatic cytolysis, histology assays, and TEM in comparison to silymarin on an animal model of thioacetamide (TAA)-induced toxic hepatitis. TAA administration determined hepatotoxicity, as it triggered redox imbalance, increased proinflammatory cytokine levels and alanine aminotransferase (ALAT) activity, and induced morphological and ultrastructural changes characteristic of liver fibrosis. In rats treated with NPCS-VL, all these pathological processes were attenuated, suggesting a potential antifibrotic effect of this hybrid bionanomaterial.

## 1. Introduction

Chronic liver disease (CLD) continues to be an important cause of morbidity and mortality worldwide, despite the fact that its pathogenesis has been intensely studied. The only curative treatment—liver transplantation—although effective, has limited availability. Hence, liver disease is among the 20 leading causes of disability-adjusted life years (DALYs) and years of life lost globally [1,2].

Different aggressors can trigger the development of CLD. Thus, in Western, industrialized countries, chronic alcohol consumption and metabolic diseases account for most cases, while in developing countries, viral hepatitis (hepatitis B virus—HBV or hepatitis C virus—HCV) still has the highest prevalence [1,3,4].

Persistent liver damage results in the replacement of functional parenchyma with fibrous connective tissue, a process called fibrosis. The key cells involved in this process are the hepatic stellate cells (HSCs) [5]. Upon activation by different factors, such as immune cells, damaged hepatocytes, inflammatory cytokines, or reactive oxygen species (ROS), these cells proliferate and trans-differentiate into collagen-producing myofibroblasts. The consecutive disruption of liver architecture can be well appreciated histologically using histochemical stains, which show collagen fibers, diffuse fibrosis, and regenerative nodules [6], and also by using transmission electron microscopy (TEM), which can display megamitochondria and Mallory–Denk bodies inside hepatocyte cytoplasm [7]. It is important to mention that, with appropriate treatment, liver fibrosis can be initially reversible. However, cirrhosis, the final stage of fibrosis, is permanent and can cause severe complications, including liver dysfunction [3]. Moreover, cirrhosis puts patients at high risk of developing hepatocellular carcinoma [8] with a mostly negative prognosis.

The progression of liver disease occurs as a result of continuous inflammation, destruction, and regeneration of hepatic parenchyma. Hepatocytes play an important role in initiating and perpetuating inflammation given their ability to release mediators (such as tumor necrosis factor—TNF, interleukin 6—IL-6, IL-1β, reactive oxygen species) and damage-associated molecular patterns (DAMPs) in response to various triggers [9,10,11]. Moreover, damaged hepatocytes release alanine aminotransferase (ALAT) and aspartate aminotransferase (ASAT), both widely used in clinical practice as reliable indicators of liver cell injury [12].

Hepatic immune cells, especially Kupffer cells, are essential in maintaining liver homeostasis. During the progression of fibrosis, they detect DAMPs and pathogen-associated molecular patterns (PAMPs) and subsequently releases cytokines and chemokines. These molecules activate HSC and other cells, therefore amplifying fibrogenesis [13,14]. Similarly, oxidative stress has been directly linked to liver fibrogenesis: ROS released by damaged hepatocytes or synthesized via NADPH oxidase (NOX) can activate nuclear factor-κB (NF-κB) or interfere with the transforming growth factor beta (TGF-β) pathway, a profibrotic cytokine which additionally stimulate ROS production [15,16].

Considering the implications of redox imbalance and inflammation in the initiation and perpetuation of liver disease, targeting these mechanisms has already been proposed with the purpose of restoring liver architecture [17]. Many natural agents have demonstrated antifibrotic properties. Thus, silymarin prevents the formation of TNF-α and other mediators [18], whereas resveratrol decreases the expression of various pro-inflammatory cytokines and promotes antioxidant enzyme activities [19]. However, when administered orally, their benefits are diminished by their low absorption and reduced bioavailability [15]. In order to overcome these limitations, nanotechnology could be an efficient tool given its ability to transport molecules in target areas [20]. Nanomaterials have a high affinity for the liver due to its rich vascularization and intense phagocytic activity of specialized cells, which makes them particularly useful in hepatic disease therapy [21]. Several studies have already demonstrated their antifibrotic, anti-inflammatory, and antioxidant properties in experimentally induced liver hepatopathy [22,23].

Our study aimed to evaluate the effects of a hybrid material (NPCS-VL) obtained by mixing gold nanoparticles capped with polyphenols from *Cornus sanguinea* L. extract (NPCS) with blueberry fruit extract (VL) on an experimental model of thioacetamide (TAA)-induced hepatopathy. The beneficial anti-inflammatory and antioxidant properties of this mixture on HSC exposed to TGF-β were already proven in a previous in vitro study [24]. Additionally, TAA has been intensively used in experimental animal models to induce hepatopathy. After metabolization in the liver, two hepatotoxic compounds, TAA-S-oxide (TAA-SO) and TAA-S-dioxide (TAA-SO_2_) are released. The metabolites themselves act as ROS, inducing lipid peroxidation in the hepatocellular membrane and promoting inflammation by increasing IL-1β, IL-6, TNF-α, and TGF-β1 secretion. It was demonstrated that TAA-triggered hepatopathy was the best model to assess the antioxidant and antifibrotic properties of various compounds in animals [25]. Based on these data, the effect of the hybrid bionanomaterial (NPCS-VL) was evaluated in comparison to silymarin on an animal model with experimental hepatopathy induced by TAA administration. Oxidative stress, inflammation markers, and histopathological and ultrastructural analyses were performed in liver samples, and hepatocyte lysis enzymes were measured in blood.

## 2. Materials and Methods

### 2.1. Vegetal Material and Reagents

Fresh *Vaccinium myrtillus* L. fruits, purchased from a local market in Cluj-Napoca (Romania), and *Cornus sanguinea* L. fruits, collected from Stâna de Mureş (46°25′31″ N; 23°59′50″ E, Transylvania, Romania), were washed with tap water, then frozen and stored until further use.

Silymarin extract (Sil) was obtained from *Silybum marianum* Gaertner and was provided by Wörwag Pharma (Böblingen, Germany). The extract had a 44.7% silymarin content expressed as silibinin [26]. Thioacetamide, o-phthalaldehyde, xanthine, xanthine oxidase, Bradford reagent, Folin–Ciocalteu reagent, sodium hydroxide, and tetrachloroauric acid were procured from Sigma–Aldrich Chemicals GmbH Inc. (Seelze, Germany), whereas 2-thiobarbituric acid and EDTA-Na2 were obtained from Merck KGaA (Darmstadt, Germany). ELISA kits for IL-6, IL-1β, TNF-α, and TGF-β levels were purchased from Elabscience (Houston, TX, USA).

The two fruit extracts were prepared by mixing 5 g of mashed fruits with 100 mL distilled water and then stirring the mixture for 60 min at room temperature. The removal of the fruit residue was achieved by vacuum filtration, and the resultant extracts were stored in the dark at 2 °C and later used for the synthesis of gold nanoparticles (CS extract) for the in vivo experiments.

The colorimetric assay using the Folin–Ciocalteu reagent was used for the quantitative determination of the total phenols in the CS and VL extracts applying a previously reported method [24]. Briefly, 3 mL of Folin–Ciocalteu reagent were added to 0.5 mL of fruit extract. After a 5 min incubation of the sample in the dark, 2.4 mL of 0.7 M sodium carbonate solution was added, and the absorbance of the resulting sample was measured two hours after, at 760 nm. The obtained value was used to calculate the total phenol content of the fruit extract using a calibration curve of the standard (gallic acid), the result being expressed as μg GAE/mL extract.

### 2.2. NPCS Preparation and Characterization

The gold nanoparticles (NPCSs) were biosynthesized using the *Cornus sanguinea* L. fruit extract as an eco-friendly reducing and stabilizing agent of the Au^3+^ from a 1 mM HAuCl_4_ solution. The CS extract (pH = 7.5, adjusted with NaOH solution) was mixed with boiling HAuCl_4_ solution in a 1:5 *v*/*v* ratio and stirred for 30 min without heating. The obtained NPCS dispersion was centrifuged in order to purify the gold nanoparticles. To remove the excess fruit extract and the unreacted gold ions, the gold pellet was washed twice with double distilled water and centrifuged again for 30 min at 12,000× *g*, using a Hettich Mikro 220 R centrifuge(Andreas Hettich GmbH & Co. KG, Tuttlingen, Germany).

The optical properties of the resulting NPCS were spectrophotometrically determined using a double beam PerkinElmer Lambda 25 UV–vis spectrophotometer (PerkinElmer Inc., Waltham, MA, USA), while their size and morphology were investigated by transmission electron microscopy (TEM) using an H-7650 120 kV Automatic TEM microscope (Hitachi, Tokyo, Japan). The polydispersity index (PDI) of the synthesized nanoparticles was determined by Dynamic Light Scattering using a Zetasizer Nano ZS-90 instrument (Malvern Instruments Ltd., Malvern, UK).

### 2.3. Experimental Animal Model

The experiment was carried out on 56 healthy, adult, male Wistar rats weighing 250 ± 20 g. All animals were obtained from the Animal Department of “Iuliu Hatieganu” University of Medicine and Pharmacy Cluj-Napoca, Romania. The animals were housed in an appropriate environment with a constant temperature of 25 °C, 35% humidity, and 12 h light/dark cycles and received a standard diet and free access to water. All experiments were approved by the Ethics Committee of “Iuliu Hatieganu” University of Medicine and Pharmacy Cluj-Napoca and were performed in accordance with ARRIVE guidelines and with the European Directive 2010/63/UE regarding the protection of the animals used for scientific purposes (no 30404.04.2022).

**Figure 1:**
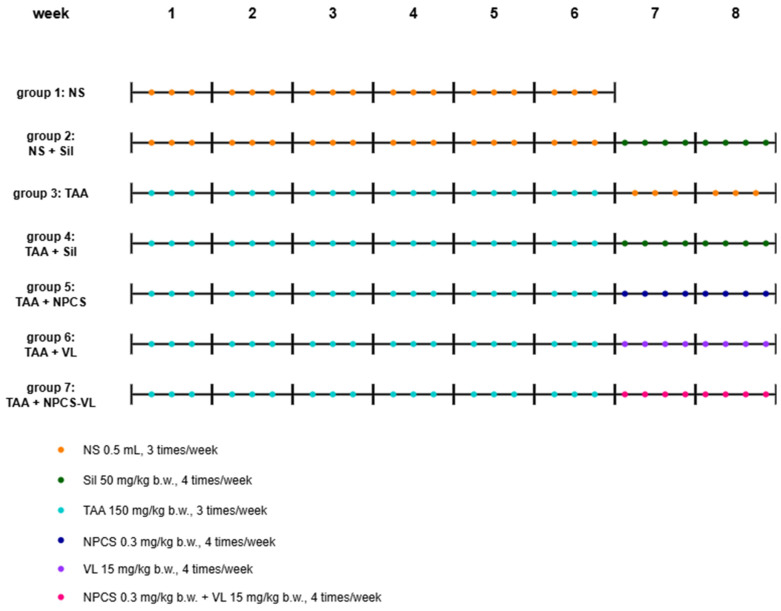
Experimental design of the study. NS—normal saline; Sil—silymarin; TAA—thioacetamide; NPCS—gold nanoparticles capped with *Cornus Sanguinea* L. extract; VL—*Vaccinium myrtillus* L. extract.

The rats were randomly divided into 7 study groups (*n* = 8), as follows (Figure 1): the control group (NS) received the vehicle—0.5 mL normal saline (NS) for 6 weeks; the NS + Sil group received 0.5 mL normal saline for 6 weeks + silymarin 50 mg/kg body weight (b.w.) for 2 weeks; the TAA group received thioacetamide 150 mg/kg b.w. dissolved in saline for 6 weeks + vehicle for another 2 weeks; the TAA + Sil group received thioacetamide 150 mg/kg b.w. dissolved in saline for 6 weeks + silymarin 50 mg/kg b.w. for 2 weeks; the TAA + NPCS group received thioacetamide 150 mg/kg b.w. dissolved in saline for 6 weeks + gold nanoparticles capped with *Cornus sanguinea* extract 0.3 mg/kg b.w. for 2 weeks; the TAA + VL received thioacetamide 150 mg/kg b.w. dissolved in saline for 6 weeks + blueberry extract (VL) 15 mg/kg b.w. for 2 weeks; and the TAA + NPCS-VL group received thioacetamide 150 mg/kg b.w. dissolved in saline for 6 weeks + the hybrid material NPCS-VL obtained by mixing 0.3 mg/kg b.w. NPCS with 15 mg/kg b.w. VL for 2 weeks. All administered substances were dissolved in saline in such a way that each animal received a total of 0.5 mL solution daily. NS and TAA were administered orally three times a week, while the natural compounds (Sil, NPCS, VL and NPCS-VL) were given by oral gavage four times per week. The standard therapeutic dose of silymarin (50 mg/kg b.w.) was chosen in accordance with similar studies of experimentally induced liver disease [27,28,29]. The doses of NPCS and VL were selected based on previous results of a cell viability assay on hepatic stellate cells [24].

Following treatments, the animals were euthanized via cervical dislocation under anesthesia with 90 mg/kg b.w. ketamine and 10 mg/kg b.w. xylazine. Liver tissue fragments were collected for biochemical assays (oxidative stress, inflammation), histopathological analysis, and electron microscopy evaluation, whereas blood samples were taken for liver enzyme quantification. The protein content in liver homogenates was measured using the Bradford method [30].

### 2.4. Oxidative Stress Quantification

Oxidative stress was quantified by malondialdehyde (MDA) and glutathione level measurement in parallel with superoxide dismutase (SOD) and glutathione peroxidase (GPx) activities evaluation. MDA levels were measured using Conti et al.’s fluorometric method with 2-thiobarbituric acid, lightly modified [31]. The activity of the antioxidant enzyme SOD was assessed via the cytochrome c reduction test [32]. The results were expressed as U/mg protein. GPx was evaluated via Flohe and Gunzler’ method, slightly adapted [33], and the enzymatic activity was expressed as micromoles of NADP produced per minute per milligram of protein. Reduced glutathione (GSH) and oxidized glutathione (GSSG) were analyzed using the fluorometric method with o-phthalaldehyde [34]. GSH/GSSG ratio was calculated and used as a marker of endogen antioxidant levels.

### 2.5. Inflammation Markers Assessment

IL-6, IL-1β, TNF-α, and TGF-β were analyzed in liver samples as markers of nonspecific inflammation using commercial ELISA Kits (Elabscience, Houston, TX, USA) according to manufacturer’s instructions. Absorbance was read at 450 nm on a Tecan Sunrise reader. The results were expressed as pg/mg protein.

### 2.6. Liver Damage Markers

To assess liver function, the blood levels of ALAT and ASAT, both used as indicators of hepatocyte lysis, were quantified. A semi-automatic method with colorimetric assay kits was applied according to manufacturer’s instructions [35], and the results were expressed as international units/liter (IU/L).

### 2.7. Transmission Electron Microscopy

Liver samples, measuring about 1 mm^3^, were prefixed for 2 h with 2.7% glutaraldehyde (Agar Scientific Ltd., Stansted, UK) in a 0.1 M phosphate buffer (pH = 7.4) at 4 °C, washed 4 times with the same buffer for 3 × 1 h and 1 overnight, and postfixed for 1.5 h with 1.5% OsO4 (Sigma-Aldrich, St. Louis, MO, USA) in 0.15 M phosphate buffer (pH = 7.4) at 4 °C. They were then dehydrated in acetone series (30–100%) and infiltrated with EMbed 812 (Electron Microscopy Sciences, Hatfield, PA, USA). From the polymerized blocks, sections of 60–80 nm were cut with a DiATOME diamond knife (DiATOME, Hatfield, PA, USA) on an LKB Ultrotome III Bromma 8800 ultramicrotome (LKB Produckter AB, Stockholm-Bromma, Sweden). These sections were collected on 300 mesh Cu grids (Agar Scientific Ltd., Stansted, UK) and contrasted for 15 min with 13% uranyl acetate and 5 min with 2.8% lead citrate. A JEOL JEM 100CX II microscope (Jeol Ltd., Tokyo, Japan) at 80 kV was used for sample examination, and the photographs were taken with a MegaView G3 camera (EMSIS, Münster, Germany).

### 2.8. Histopathological Analysis

For histopathology analysis, the liver fragments were fixed for 24 h in a 10% phosphate-buffered formalin solution and then embedded in paraffin wax. The samples were cut into 5 μm sections using a Reichert microtome (Vienna, Austria) and later stained with hematoxylin and eosin (H&E) and Van Gieson. Slides were examined by two independent specialists without previous knowledge of the experimental protocol using an Olympus BX51 microscope (Tokyo, Japan).

### 2.9. Statistical Analysis

The statistical analysis was performed with one-way ANOVA followed by Tukey’s posttest using GraphPad Prism version 5.00 for Windows (GraphPad Software, San Diego, CA, USA, www.graphpad.com). All mentioned data were expressed as mean ± standard deviation (SD), with a *p* value < 0.05 being considered statistically significant.

## 3. Results

### 3.1. Fruit Extracts and NPCS Characterization

The fruit extracts were characterized by total polyphenol content (TPC) measurement using the Folin–Ciocalteu method. The CS extract contained a large number of phenolic compounds (TPC = 326.34 ± 12.91 μg GAE/mL extract), which enabled us to use it for the biosynthesis of the gold nanoparticles.

The high TPC of the fruit extract is correlated with a high reducing power of the phenolic compounds, which are able to donate electrons of their hydroxyl groups to reduce Au^3+^ ions to elemental gold, initiating the formation of the gold nanoparticles. The presence of the phenolic content directly affects the rate, yield, and characteristics (size and shape) of the synthesized metallic nanoparticles. A higher concentration of polyphenols in the extract often results in a faster nucleation, leading to smaller nanoparticles [36].

After mixing the fruit extract with the Au^3+^ solution, the reducing reaction took place as indicated by the rapid color change from light red to intense purple.

**Figure 2:**
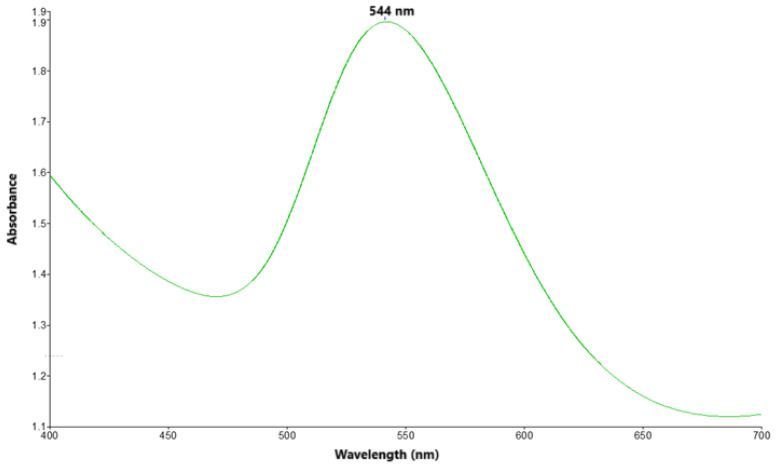
UV–vis spectrum of NPCSs.

UV–vis spectroscopy and transmission electron microscopy (TEM), two powerful tools for gold nanoparticles’ characterization, were performed. The UV–vis spectrum of the NPCSs (Figure 2) showed a characteristic Surface Plasmon Resonance (SPR) band at 544 nm, thus confirming their formation. Spherical NPCSs exhibited a strong SPR band typically in the range 520–550 nm [37].

**Figure 3:**
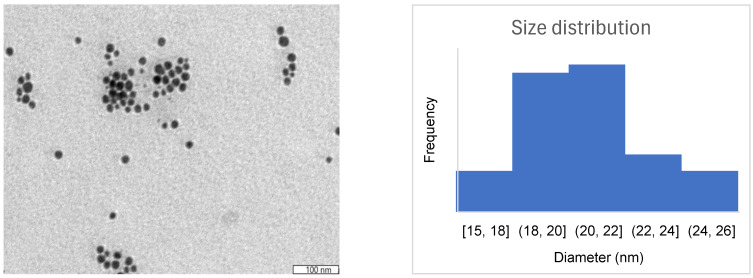
The morphology and size distribution of NPCSs evaluated by TEM.

TEM investigated the structural properties of the nanomaterials by providing detailed information on their size, shape, and surface morphology. The TEM image of the *Cornus sanguinea* L. extract-mediated gold nanoparticles (Figure 3) shows that the obtained nanoparticles were mostly spherical, with an average diameter of 20 nm, confirming the results obtained by the UV–vis spectroscopy.

The polydispersity index of the synthesized gold nanoparticles was determined by Dynamic Light Scattering (DLS) in order to investigate the width of the size distribution of the particles in the colloidal suspension. The obtained PDI value of the synthesized gold nanoparticles was 0.258, indicating a moderately polydisperse nanoparticles sample with minor variation in particle sizes, but likely containing a main population of particles. The size distribution of the synthesized gold nanoparticles, as measured by DLS using intensity weighting, revealed a main population of NPCSs (95.2%) having a hydrodynamic diameter of 537 nm and a secondary peak (4.8%) at ~92 nm.

### 3.2. Oxidative Stress Evaluation

**Figure 4:**
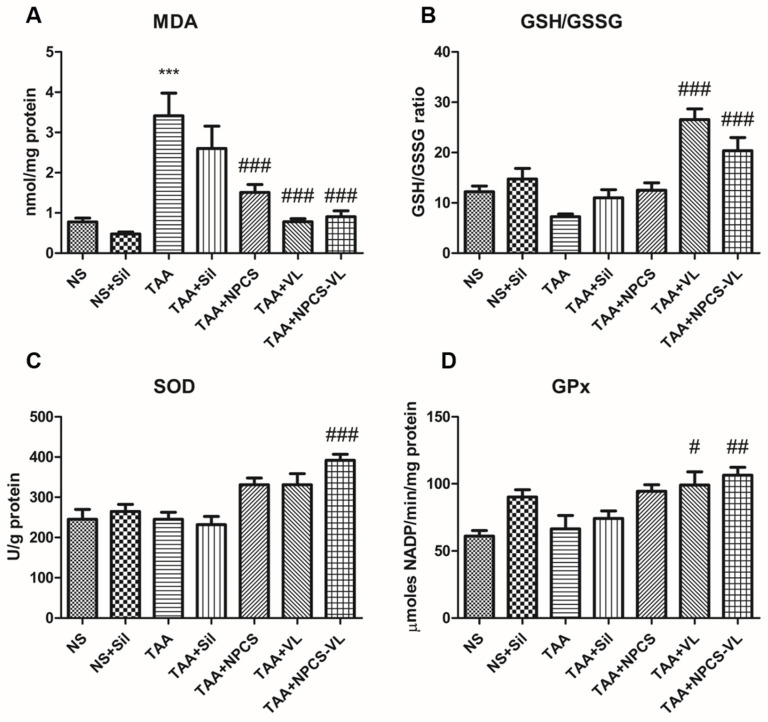
Oxidative stress markers evaluation in rat liver tissue exposed to TAA and treated with silymarin, NPCS, VL, or NPCS-VL. (**A**) Malondialdehyde levels enhanced after TAA administration (*p* < 0.001), effect counteracted by NPCS, VL, and NPCS-VL (*p* < 0.001). (**B**) GSH/GSSG ratio improved after treatment with VL and NPCS-VL (*p* < 0.001). (**C**) SOD activity in liver samples was amplified after NPCS-VL (*p* < 0.001). (**D**) GPx activity increased in liver fragments exposed to VL (*p* < 0.05) and NPCS-VL (*p* < 0.01). Data are expressed as means ± standard deviations, and the results are expressed as nmoles, U, or μmoles per mg protein in tissue homogenates. The statistical significance of the difference between the treated and control group was evaluated with one-way ANOVA followed by Tukey posttest, *** *p* < 0.001 vs. the NS group and # *p* < 0.05, ## *p* < 0.01, and ### *p* < 0.001 vs. the TAA group. MDA—malondialdehyde; GPx—glutathione peroxidase; GSH/GSSG—reduced glutathione/oxidized glutathione; NPCSs—gold nanoparticles capped with *Cornus Sanguinea* L. extract; NS—normal saline; Sil—silymarin; SOD—superoxide dismutase; TAA—thioacetamide; VL—*Vaccinium myrtillus* L. extract.

In order to evaluate the redox status and MDA as a lipid peroxidation marker, non-enzymatic (GSH/GSSG ratio) and enzymatic antioxidant (SOD, CAT, and GPx) activities were assessed from liver homogenates. Thioacetamide administration augmented MDA levels compared to the control group, indicating its prooxidant properties. This effect was counteracted by treatment with VL, NPCS, and their mixture (*p* < 0.001). VL and NPCS-VL increased the GSH/GSSG ratio (*p* < 0.001) and GPx activity (*p* < 0.05) after TAA administration, suggestive the free radical scavenging effect of these compounds. Concerning SOD activity, which acts as an antioxidant defense, only NPCS-VL amplified it significantly (*p* < 0.001) in animals exposed to TAA (Figure 4).

### 3.3. Inflammation Markers Assessment

**Figure 5:**
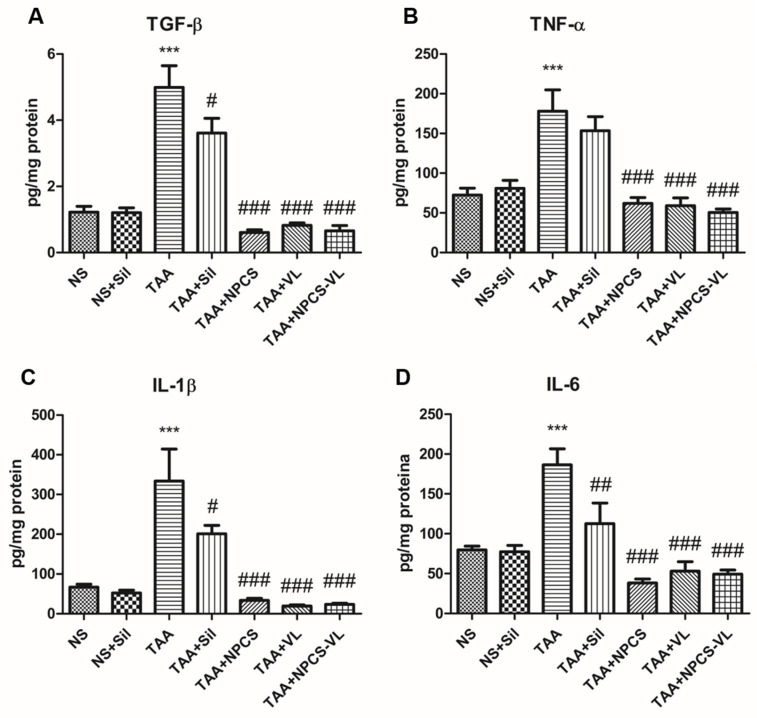
Proinflammatory cytokines quantification in rat liver tissue exposed to TAA and treated with silymarin, NPCS, VL, or NPCS-VL. TAA significantly increased all inflammation markers’ levels in liver samples (*p* < 0.001). (**A**) TGF-β levels decreased after Sil (*p* < 0.05), NPCS, VL, and NPCS-VL administration (*p* < 0.001). (**B**) NPCS, VL, and NPCS-VL administration counteracted TAA’s effects on TNF-α levels (*p* < 0.001). (**C**) IL-1β expression diminished in samples treated with Sil (*p* < 0.05) and NPCS, VL, and NPCS-VL (*p* < 0.001). (**D**) IL-6 secretion was reduced by Sil (*p* < 0.01), NPCS, VL, and NPCS-VL (*p* < 0.001). Data are expressed as means ± standard deviations, and the results are expressed as pg cytokine per mg protein in tissue homogenates. The statistical significance of the difference between treated and control group was evaluated with one-way ANOVA, followed by Tukey posttest, *** *p* < 0.001 vs. NS group and # *p* < 0.05, ## *p* < 0.01, and ### *p* < 0.001 vs. TAA group. IL—interleukin; NPCS—gold nanoparticles capped with *Cornus Sanguinea* L. extract; NS—normal saline; Sil—silymarin; TAA—thioacetamide; TGF-β—transforming growth factor-β; VL—*Vaccinium myrtillus* L. extract.

Thioacetamide promoted inflammation in liver tissue, increasing TGF-β, TNF-α, IL-1β, and IL-6 levels in liver homogenates. This effect was strongly reversed by NPCS, VL, and NPCS-VL administration, thus demonstrating that these natural compounds have anti-inflammatory properties (*p* < 0.001). Silymarin also decreased TGF-β (*p* < 0.05), IL-1β (*p* < 0.05), and IL-6 (*p* < 0.01) secretion (Figure 5) in animals exposed to TAA, suggesting its hepatoprotective properties. However, NPCS, VL, and NPCS-VL exhibited stronger anti-inflammatory effects than Silymarin. TGF-β (*p* < 0.001), TNF-α (*p* < 0.001), IL-1β (*p* < 0.01), and IL-6 (*p* < 0.05) all had lower levels in rats treated with NPCS, VL, or NPCS-VL than in animals which received Silymarin.

### 3.4. Liver Damage Markers

**Figure 6:**
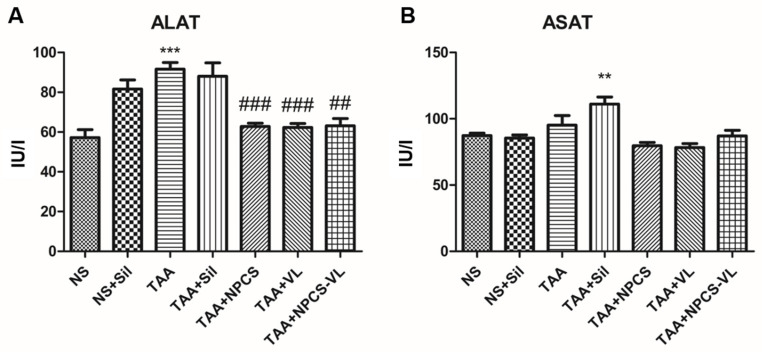
Serum activities of ALAT and ASAT in rats exposed to TAA and treated with silymarin, NPCS, VL, or NPCS-VL. (**A**) TAA significantly increased serum ALAT levels (*p* < 0.001), while NPCS, VL, and NPCS-VL administration all ameliorated this effect (*p* < 0.001). (**B**) The effects of TAA, Sil, NPCS, VL, or NPCS-VL administration on ASAT activity were not statistically significant. Data are expressed as means ± standard deviation, and the results are expressed as IU/l. The statistical significance of the difference between the treated and control group was evaluated with one-way ANOVA followed by Tukey posttest, ** *p* < 0.01; *** *p* < 0.001 vs. NS group; and ## *p* < 0.01, ### *p* < 0.001 vs. TAA group. ALAT—alanine aminotransferase; ASAT—aspartate aminotransferase; NPCSs—gold nanoparticles capped with *Cornus Sanguinea* L. extract; NS—normal saline; Sil—silymarin; TAA—thioacetamide; VL—*Vaccinium myrtillus* L. extract.

To assess the liver function, ALAT and ASAT blood levels were measured. ALAT is more specific to liver damage, and its activity increased after TAA administration, demonstrating TAA’s ability to induce hepatic cytolysis. This liver damage was significantly ameliorated by NPCS, VL, and NPCS-VL administration, suggestive for their hepatoprotective properties (Figure 6A). ASAT activity was increased when TAA and silymarin were co-administered, but the other natural agents did not significantly influence its levels (Figure 6B).

### 3.5. Histopathology Analysis

**Figure 7:**
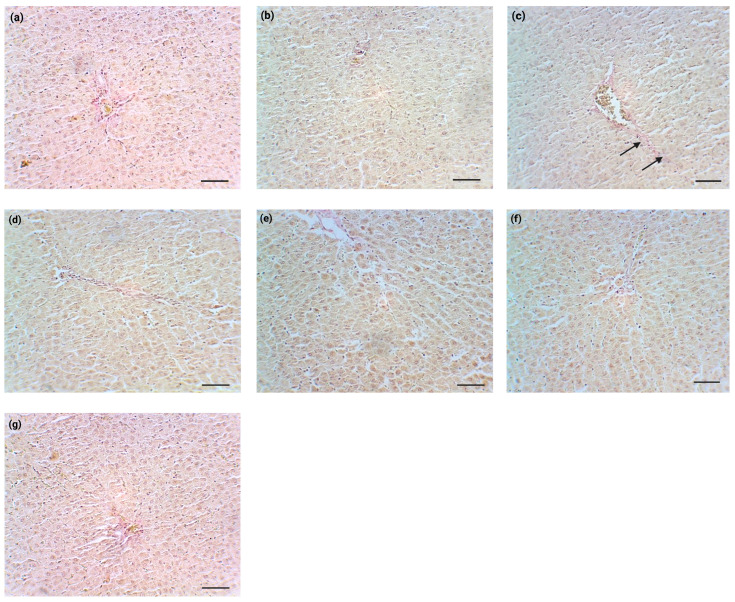
Histopathological aspects of the liver treated with normal saline (**a**), silymarin (**b**), TAA, (**c**) TAA and silymarin (**d**), NPCS (**e**), VL (**f**), or NPCS-VL (**g**). Perivascular collagen deposits were highlighted by Van Gieson staining, which stained these deposits pink-fuchsia. TAA group (**c**) showed a greater collagen accumulation compared to the control group (**a**) and normal saline + silymarin group (**b**). Treatments with silymarin, NPCS, VL, and NPCS-VL reduced the pre-fibrotic aspects of the liver determined by TAA exposure, thus being potential preventive strategies in the pathology of hepatic fibrosis (**d**–**g**). Magnification × 200, scale bar = 25 µm.

Samples collected from the NS and Sil groups showed normal liver histology. The hepatocytes did not develop hydropic or granular degeneration, and cholestatic signs were not found. Moreover, liver infiltration with mononuclear cells was not identified, which suggests that liver responses to treatments were restricted to fibrosis-like changes with thin collagen deposition near the liver vascular network. Thus, TAA promoted morphological changes characteristic of liver fibrosis, mainly portal and periportal collagen deposition. Sil, NPCS, VL, or NPCS-VL administration ameliorated TAA-induced alterations in liver architecture (Figure 7).

### 3.6. Ultrastructural Investigation

**Figure 8:**
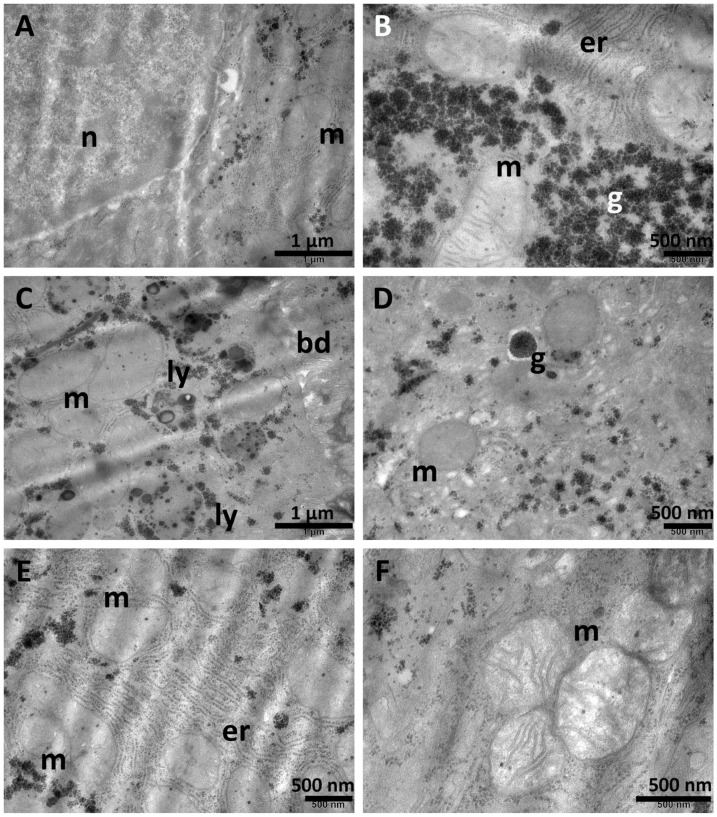
Normal ultrastructural aspects of the liver tissue in the NS group (**A**,**B**) and Sil group (**C**–**F**) showing nuclei (**A**), endoplasmic reticulum (**B**,**E**), numerous mitochondria (**A**–**F**), and glycogen granules (**B**,**D**), as well as occasional lysosomes (**C**). A normal bile canaliculus is observed in (**C**). bd—bile canaliculus; er—endoplasmic reticulum; g—glycogen; ly—lysosomes; m—mitochondria; n—nucleus. Scale bar = 500 nm–1 µm.

TEM examination of hepatic tissue fragments from both the NS group and Sil group (Figure 8) showed normal architecture of the liver. The hepatocytes contained one or two large, spherical nuclei (Figure 8A) with prominent nucleoli, cytoplasm with an important amount of endoplasmic reticulum (Figure 8B,E), rare small lysosomes (Figure 8C), oval mitochondria, and numerous glycogen granules (Figure 8B,D). Normal bile canaliculi were observed between adjacent hepatocytes (Figure 8C).

**Figure 9:**
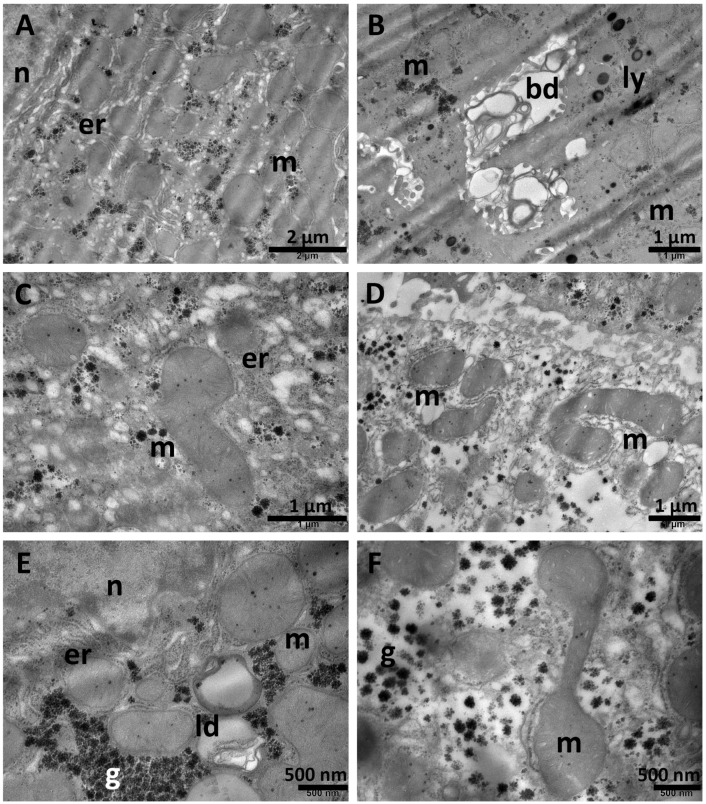
Ultrastructural aspects of the liver tissue in the TAA group. Some nuclei preserve their rounded shape (**A**), while others are irregular (**E**). Expanded profiles of endoplasmic reticulum are noted (**A**,**C**), as well as long and polymorphic mitochondria (**C**,**D**,**F**). Numerous glycogen granules are present (**E**), alongside heterogenous lipid droplets (**E**). A dilated bile canaliculus is shown in (**B**). bd—bile canaliculus; er—endoplasmic reticulum; g—glycogen; ld—lipid droplets; ly—lysosomes; m—mitochondria; n—nucleus. Scale bar = 500 nm–2 µm.

TAA administration induced several ultrastructural changes. While some nuclei preserved their round shape (Figure 9A), others had an irregular contour (Figure 9E). Inside the cytoplasm, the endoplasmic reticulum was proliferated and expanded, and the mitochondria were long and polymorphic (Figure 9F). Large, heterogenic lipid droplets were noted (Figure 9E), as well as abundant glycogen granules (Figure 9F). Some bile canaliculi were dilated, with a large amount of precipitate inside (Figure 9B). Figure 9D,F show typical aspects of necrotic hepatocytes with severe architectural alterations.

**Figure 10:**
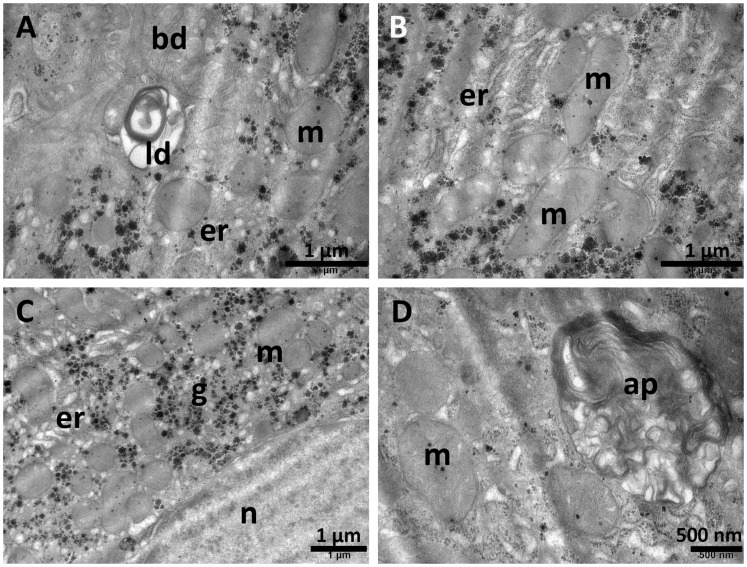
Ultrastructural aspects of the liver tissue in the TAA + Sil group. Large and heterogenous lipid droplets are observed (**A**), mitochondria with aberrant aspects are present (**B**,**C**) and the endoplasmic reticulum is enlarged (**C**). Autophagosomes are also noted (**D**). ap—autophagosome; bd—bile canaliculus; er—endoplasmic reticulum; g—glycogen; ld—lipid droplets; m—mitochondria; n—nucleus. Scale bar = 500 nm–1 µm.

In the TAA + Sil group, the endoplasmic reticulum was still enlarged (Figure 10C), and a few mitochondria displayed aberrant shapes (Figure 10B,C). The lipid droplets were large and heterogenic (Figure 10A), and the glycogen had normal distribution (Figure 10A–C). Also, the numerous and large autophagosomes contained multiple whorls of membranous structures (Figure 10D). Most of the observed nuclei showed a normal aspect (Figure 10C).

**Figure 11:**
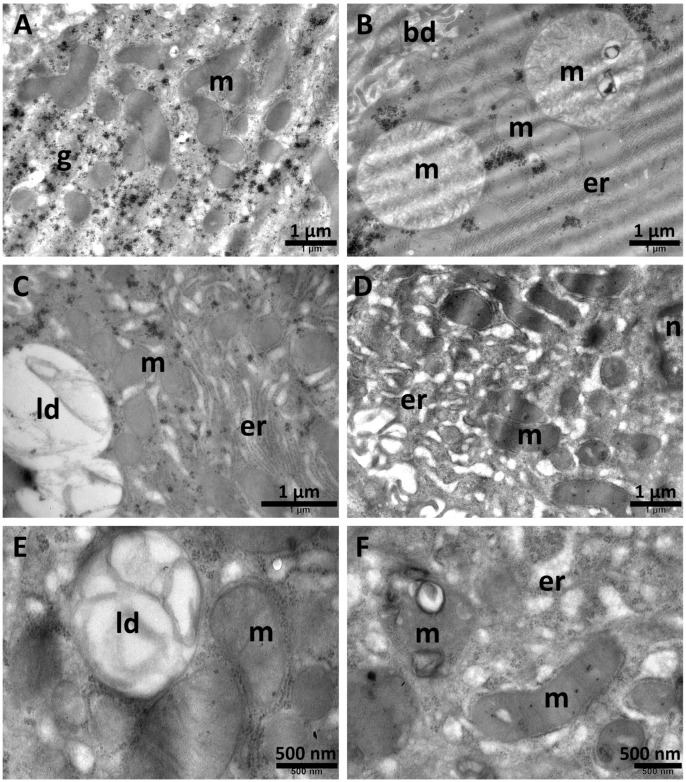
Ultrastructural aspects of the liver tissue in the TAA + NPCS group. Numerous affected mitochondria are present (**A**–**F**), as well as expanded profiles of endoplasmic reticulum (**A**,**C**,**D**,**F**). The lipid droplets contain dense inclusions (**C**,**E**). bd—bile canaliculus; er—endoplasmic reticulum; g—glycogen; ld—lipid droplets; m—mitochondria; n—nucleus. Scale bar = 500 nm–1 µm.

In the hepatocytes of the group treated with TAA + NPCS, the mitochondria remained among the most affected cellular structures, sometimes on a background of a rarefied cytoplasm. Thus, many were polymorphic (Figure 11A,C–F); others were large and swollen, with an electron–lucent matrix (Figure 11B) and/or with abnormal cristae (Figure 10B,F). Additionally, in numerous mitochondria, vesicles with heterogeneous content were found (Figure 11B,F). The second most affected organelle in this group was the endoplasmic reticulum. It was mostly expanded, contributing to the general rarefaction of the cytoplasm (Figure 11A,C,D,F). All the lipid droplets found were large and with dense inclusions (Figure 11C,E). Glycogen was scarce in the hepatocytes of this group.

**Figure 12:**
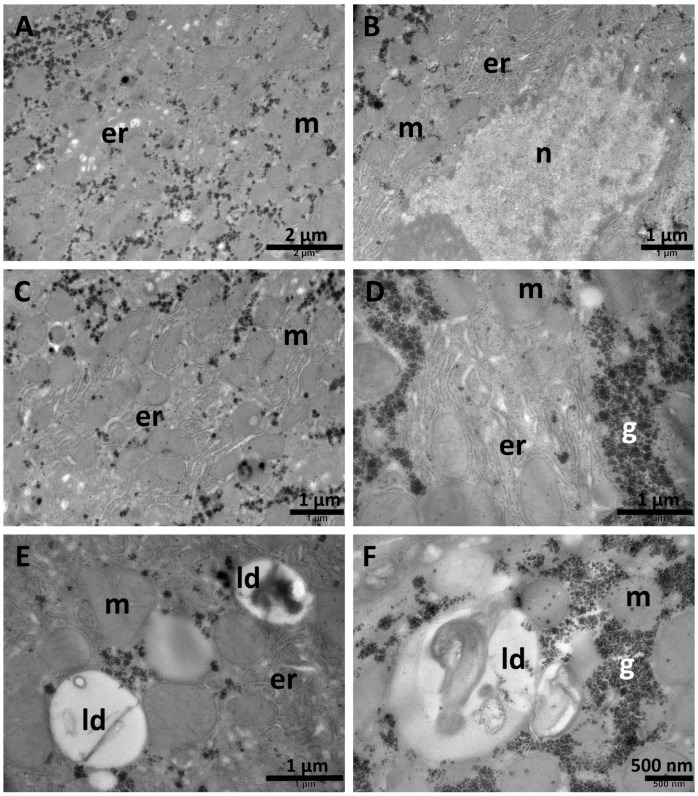
Ultrastructural aspects of the liver tissue in the TAA + VL group. Some nuclei have irregular contour (**B**), and in some areas, the endoplasmic reticulum is expanded (**A**,**C**,**D**). Heterogenous lipid droplets are also present (**E**,**F**). er—endoplasmic reticulum; g—glycogen; ld—lipid droplets; m—mitochondria; n—nucleus. Scale bar = 500 nm–2 µm.

In the hepatocytes of the TAA + VL group, the endoplasmic reticulum was normal or slightly expanded in some areas (Figure 12A–E). Occasional polymorphic mitochondria were observed (Figure 12A–C,E), as well as abundant glycogen granules scattered in the cytoplasm (Figure 12A–F). The lipid droplets were large, mostly heterogenous, containing various electron-dense inclusions (Figure 12E,F). The nuclei of some cells had an irregular contour (Figure 12B).

**Figure 13:**
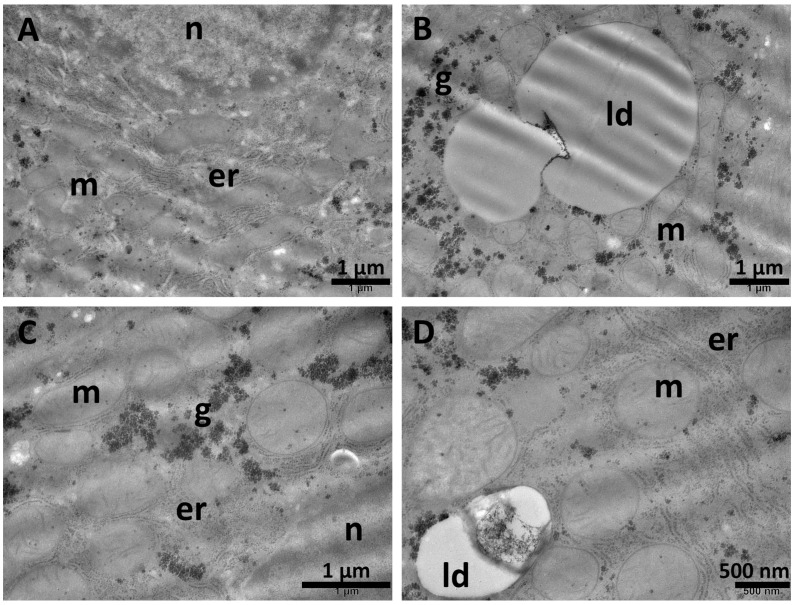
Ultrastructural aspects of the liver tissue in the TAA + NPCS-VL group. The endoplasmic reticulum is slightly expanded (**A**), and occasional polymorphic mitochondria are present (**A**,**B**,**C**). The lipid droplets are large and homogenous (**B**) or contain dense inclusions (**D**). er—endoplasmic reticulum; g—glycogen; ld—lipid droplets; m—mitochondria; n—nucleus. Scale bar = 500 nm–1 µm.

In the TAA + NPCS-VL group, the nuclei of most hepatocytes were round-oval with regular contour (Figure 13A). In the cytoplasm, a slight dilation of the endoplasmic reticulum (Figure 13A), large and homogeneous (Figure 13B) or heterogeneous (Figure 13D) lipid droplets, and occasional polymorphic mitochondria (Figure 13A,B) were observed. Apart from these modifications, the liver architecture was approximately normal.

## 4. Discussion

Over the past years, the interest in using plant-based products for the prevention and treatment of liver disease has grown significantly. Their low toxicity together with their ability to interfere with the pathogenetic mechanisms that lead to fibrogenesis make these compounds promising therapeutic agents. Among herbal remedies, silymarin, resveratrol, and curcumin were the most intensely studied. Rich in bioactive compounds such as flavonoids, these natural extracts have proven their antifibrotic properties in various studies of experimentally induced liver injury via modulation of ROS production, inflammation, and collagen synthesis [38,39].

The used antifibrotic agents have limited efficiency in patients as a result of their lack of specificity for the target organ, which sometimes leads to contradictory effects. For example, in vitro, interferon gamma (IFN-γ) stimulates HSC apoptosis, consequently inhibiting fibrogenesis, while in vivo it has proinflammatory effects by activating macrophages. Nanotechnology proved useful in combating this limitation, as it facilitates using drug transport systems with high affinity to target tissues [20]. Gold nanoparticles are among the most preferred nanomaterials due to their stability, biocompatibility, low toxicity, and facile functionalization [40], especially with various polyphenols. *Cornus sanguinea* (CS) extract is abundant in bioactive molecules, among which flavonoids (e.g., quercetin, isoquercitrin, kaempferol, rutin), vitamin C, phenolic acids (e.g., caffeic, chlorogenic, ferrulic), tannins, and lignans not only show metal-reducing activity but also demonstrate antioxidant, anti-inflammatory, and antimicrobial effects [41,42]. Reported literature data supports the benefits of administering natural extracts from *Cornus* species together with nanomaterials in inflammation and liver fibrosis. Zugravu et al. demonstrated that gold nanoparticles phytoreduced with *Cornus mas* ameliorated oxidative stress, inflammation, and liver architecture in an experimental model of high-fat diet-induced hepatopathy [43]. Additionally, *Vaccinium myrtillus* L. extract is an herbal product with clear benefits in liver fibrosis treatment attributable to its content in anthocyanins, procyanidins, and other polyphenols. Recent studies have demonstrated that VL exhibited free radical scavenger and antifibrotic properties in CCl_4_-induced hepatopathy [44] and stimulated antioxidant protein synthesis following hydrogen peroxide administration in mice [45]. By combining this extract with NPCS, we aimed to obtain a hybrid compound with augmented benefits in liver fibrosis treatment.

Taking this data into account, we aimed to evaluate whether a hybrid material obtained by combining VL extract and gold nanoparticles phytoreduced with CS extract (NPCS-VL) has therapeutic benefits in TAA-induced liver injury. A previous in vitro study of this mixture showed promising outcomes, demonstrating the anti-inflammatory and antioxidant properties [24]. However, an in vivo experiment is needed in order to complete these results, as it gives the opportunity to extensively evaluate NPCS-VL’s properties on the organism as a whole. The mixture’s ability to ameliorate liver fibrosis was assessed through biochemical tests (liver oxidative stress and inflammation markers, blood aminotransferase levels), histopathological investigations, and transmission electron microscopy images.

The results showed that MDA levels increased after TAA administration, while NPCS, VL, and NPCS-VL all counteracted this effect. Treatment with VL and NPCS-VL in rats exposed to TAA augmented GSH/GSSG ratio and stimulated GPx activity, while the SOD enzyme was only increased by NPCS-VL administration. Therefore, NPCS-VL demonstrated free radical scavenger properties. Additionally, TAA enhanced TGF-β, TNF-α, IL-1β, and IL-6 secretions, whereas treating rats with NPCS, VL, and NPCS-VL after TAA administration significantly reduced proinflammatory cytokines’ levels. Silymarin had therapeutic benefits on TGF-β, IL-1β, and IL-6 secretion. ALAT activity was stimulated by TAA, and the effect diminished after NPCS, VL, and NPCS-VL treatment. Histopathology assessment of liver tissue showed portal and periportal collagen deposition after TAA administration, the most affected group being TAA + Sil, while NPCS, VL, and NPCS-VL noticeably reduced these fibrotic changes. TEM analysis confirmed that TAA induced changes in hepatocyte nuclei, endoplasmic reticulum, mitochondria, and bile canaliculi; glycogen granules and lipid droplets were also observed. Sil, NPCS, and VL administration partially attenuated these pathological aspects, while rats treated with NPCS-VL had an approximately normal ultrastructure of their liver.

It is known that CLD occurs as a response to a persistent injurious agent and is characterized by excessive ECM deposition in liver, causing architectural distortion and organ dysfunction. Myofibroblasts, a population of α-SMA-positive cells normally absent in healthy hepatic tissue, are responsible for this process and are therefore an important target in antifibrotic therapy. Most of these cells originate from quiescent HSC, which, when activated by various mediators (e.g., inflammatory cytokines, free radicals, PAMPs), adopt a contractile phenotype and synthetize large amounts of fibrillary collagens [13,46]. As inflammation and oxidative stress generation are key mechanisms in fibrosis development, our study aimed to evaluate the potential benefits of NPCS-VL on these two processes. For this purpose, liver damage was induced by TAA administration. We chose this model due to TAA’s ability to induce persistent damage, its facile administration, and its high reproducibility. Moreover, the interruption of TAA administration allowed us to study fibrosis regression [47]. This toxin’s effects occur after its oxidation to TAA-SO and TAA-SO_2_, via cytochrome P450 (CYP2E1), in centrilobular hepatocytes. These unstable metabolites act as profibrotic agents by various mechanisms, including NOX induction, lipid peroxidation in the hepatocellular membrane, inflammasome activation, NF-κB pathway stimulation with consequent inflammatory cytokine release, centrilobular hepatocyte necrosis [25,48], and some degree of ductal proliferation, similar to the lesions found in human fibrotic liver [49]. Slight periportal hepatocyte injury was also reported [22].

Our study confirmed TAA’s prooxidant and proinflammatory potential, as it increased lipid peroxidation and inflammatory cytokine synthesis (TGF-β, TNF-α, IL-1β, and IL-6). It also induced cytolysis, portal and periportal collagen deposition, hepatocyte necrosis, and various liver ultrastructural alterations (such as damaged nuclei, proliferated endoplasmic reticuli, polymorphic mitochondria, and deposition of lipid droplets and glycogen granules).

Gold nanoparticles have been widely used with both diagnostic and therapeutic purposes in liver disease due to their qualities alongside their high affinity for this organ. Amongst others, they act as biosensors in HBV or HCV detection [23], and they exhibit anti-inflammatory, antioxidant, and antifibrotic benefits in various models of experimentally induced liver injury, using alcohol and methamphetamine [50] or CCl_4_ [51] as inductors. Their formation can be achieved by different chemical, physical, or biological processes; however, the method that recently gained the most popularity is the biological one using plant extracts. This type of green synthesis is based on the reducing and stabilizing properties of phytochemicals contained in herbal products, ensuring natural, non-toxic nanoparticles [52]. In the present study, CS extract, due to its aforementioned properties, was chosen in order to achieve such nanomaterials. Following synthesis, gold nanoparticles’ characterization is essential for understanding their properties and applications, especially in bionanomedicine. UV–Vis spectroscopy and TEM provided convenient and powerful tools, certifying the appropriate formation of spherical nanomaterials, with a mean diameter of 20 nm and a UV–Vis spectrum showing a peak at λmax = 544 nm. Theoretically, spherical gold nanoparticles in solution [37] exhibit a characteristic absorption band in the range of 520–580 nm. The PDI value of 0.258 suggests that the synthesized gold nanoparticles present moderate size uniformity, being acceptable for most practical uses, like biosensing, catalysis, or drug delivery, though not highly monodisperse [53].

Oxidative stress, defined as the imbalance between ROS generation and the scavenging capacities of antioxidant systems, is a key mechanism in liver fibrogenesis. Under physiological conditions, free radicals modulate multiple cell processes, including defense against microorganisms and signal transduction; however, when present in excessive amounts, they alter cell homeostasis, resulting in hepatocyte necrosis and apoptosis, HSC activation, and profibrogenic mediators’ synthesis by KC [54]. Moreover, when insulted, all these cells produce ROS themselves, thus perpetuating liver dysfunction [55]. Targeting oxidative stress has been proposed as an approach to liver disease therapy, demonstrating satisfactory results. For example, silymarin’s antifibrotic properties result from its ability to prevent liver peroxidation and increase glutathione production [18]. NPCS-VL was synthetized from substances with proven antioxidant properties, and it successfully reduced oxidative stress, as it diminished MDA generation (an end-product of lipid peroxidation), it amplified GSH formation (resulting in an augmented GSH/GSSG ratio), and it stimulated the activity of antioxidant enzymes SOD and GPx.

Chronic inflammation and fibrogenesis are closely interconnected; once initiated, the two processes amplify each other, eventually leading to cirrhosis. The liver is rich in immune cells, able to quickly respond to injurious agents. For example, DAMPs released during hepatocyte death or PAMPs translocated into the bloodstream as a result of increased gut permeability (characteristic of liver disease) initiate a type 1 inflammatory response, with subsequent recruitment of immune cells and release of pro-inflammatory cytokines and chemokines. Interaction between DAMPs/PAMPs and their receptors stimulate inflammasome assembly, which perpetuates cytokine formation, stimulates fibrogenic genes’ expression (e.g., TGF-β, collagen type I), and activates HSC [56]. Moreover, IL-17 produced by certain populations of leukocytes upregulates TGF-β receptor II in HSC, thus increasing their sensitivity to this well-known profibrotic cytokine [57]. Silymarin is an antifibrotic drug with known anti-inflammatory properties [58]; our study demonstrated this once more. Concerning NPCS-VL, it managed to decrease the levels of all proinflammatory mediators evaluated in the present experiment.

Increased serum transaminase levels have long been used in clinical practice as indicators of a hepatocellular pattern of liver damage. While ALAT is particularly specific for liver diseases, ASAT elevation can be observed as a consequence of various injuries, including muscle and thyroid disorders [59]. NPCS, VL, and NPCS-VL attenuated TAA-induced hepatic cytolysis, demonstrating their ability to improve liver function. Similarly, Yan et al. reported that blueberry administration diminished transaminase levels in rats which previously received CCl_4_ [60]. The discordant levels of ASAT and ALAT in the TAA + Sil group could be attributed to ASAT’s lack of specificity for the liver.

Numerous data from the literature are consistent with ours and confirm the hepatoprotective effects of gold nanoparticles capped with natural extracts. Thus, Opris et al. showed that phytosynthesized gold nanoparticles using *Sambucus nigra* L. natural extract diminished inflammation and oxidative stress in the liver in animals with hyperglycemia. Furthermore, the nanoparticles did not exert supplementary hepatotoxicity, suggesting that the 20 nm size of the particles is suitable for administration as adjuvants in the treatment of liver diseases [61]. Clichici et al. demonstrated the beneficial effects of gold nanoparticles phytoreduced with silymarin in diminishing cholestasis, redox imbalance, inflammation, and liver fibrosis in an experimental model of cholestasis [27]. Besides their antioxidant activity, gold nanoparticles conjugated with curcumin reduced the collagen synthesis in NIH/3T3 cells without having a toxic effect on chicken embryos [62].

Optical (OM) and transmission electron microscopy allow for direct visualization of morphological and ultrastructural changes induced by profibrotic stimuli. Liver biopsy has long been essential in the follow-up of patients with CLD, useful for assessing the severity and progression of hepatic insult. Grading and staging take into account various features, including hepatocellular changes, biliary alterations, inflammatory response, and fibrotic transformation [63]. In our study, TAA exposure generated such liver injury, with considerable improvement after treatment with NPCS-VL. As TEM provides a more detailed image of cell structure, it enabled us to better analyze the effects of the administered compounds. The modifications induced by TAA were comparable to those described in previous studies of experimentally induced liver fibrosis with deltamethrin [64] or in patients with HCV infection [65]: nuclei with irregular shapes and altered morphology, elongated mitochondria, dilated rough ER, and large lipid droplets. NPCS-VL ameliorated these alterations, restoring liver architecture to an approximately normal aspect.

In the present study, NPCS-VL was assessed in comparison to silymarin, a natural product already in use as supportive treatment in CLD for its hepatoprotective properties [66]. While both compounds exhibited strong anti-inflammatory effects, NPCS-VL demonstrated additional benefits: it attenuated oxidative stress and ameliorated the hepatocyte lysis. Consequently, NPCS-VL could be a valuable tool in liver fibrosis management.

## 5. Conclusions

The treatment of CLD remains a challenge for clinicians, even though the reversibility of liver fibrosis is possible under certain circumstances. Targeting inflammation and oxidative stress, key mechanisms in fibrogenesis, has proven to be an efficient approach. Our results proved that NPCS-VL had free radical scavenging properties, diminished pro-inflammatory cytokines’ release, and exhibited hepatoprotective effects by reducing ALAT activity and improvement of microscopic changes. The benefits of NPCS-VL administration were highlighted through morphological assays, and the hybrid compound reduced collagen deposition and ameliorated organelle damage induced by TAA.

## Data Availability

The original contributions presented in this study are included in the article. Further inquiries can be directed to the corresponding author.
